# Exploration of the Donors and Specific Genes of B Subgenome in *Perilla frutescens* Based on Genomic Analysis

**DOI:** 10.3390/plants14233698

**Published:** 2025-12-04

**Authors:** Zhaoyuan Li, Bin Wang, Wei Wei, Yang Liu, Qiuling Wang, Zhihui Gao, Jianhe Wei

**Affiliations:** 1 State Key Laboratory of Bioactive Substance and Function of Natural Medicines & National Engineering Laboratory for Breeding of Endangered Medicinal Materials, Institute of Medicinal Plant Development, Chinese Academy of Medical Sciences & Peking Union Medical College, Beijing 100193, China; 2 Hainan Provincial Key Laboratory of Resources Conservation and Development of Southern Medicine, Hainan Branch of the Institute of Medicinal Plant Development, Chinese Academy of Medical Sciences and Peking Union Medical College, Haikou 570311, China

**Keywords:** *Perilla frutescens*, ancestor, B subgenome, asymmetric evolution, TPS, NB-ARC

## Abstract

*Perilla frutescens* is an important medicinal and edible plant in Asia and was introduced in Europe and North America mainly as a spice plant. The commonly cultivated species is an allotetraploid (AABB). While the identity of its AA diploid donor has been preliminarily clarified, the other donor, BB, has not been discovered yet, and the taxonomic status and characteristics of the BB donor remain unresolved. Based on the published genomes of *Perilla* spp., we employed a collinearity analysis, gene structure similarity assessment, and multi-level functional annotation to infer the genomic and phenotypic features of the B subgenome. Results suggest that the protein sequences of the B and A subgenomes exhibit the highest similarity, while the protein sequences of *Lavandula angustifolia* or *Ocimum basilicum* are less similar to the B subgenome, and two subgenomes also possess the largest number of homologous genes and have similar gene structures. A total of 90 BB progenitor-specific genes were significantly enriched in pathways related to secondary metabolite biosynthesis and environmental stress response. Among these genes, the terpene synthase genes constitute the main genetic basis for the diversity of bioactive components in perilla. The discovery of a homologous gene containing the NB-ARC domain, associated with resistance to late blight, suggests that BB may contribute to key disease-resistant traits. Further gene family analysis revealed that compared with the A subgenome, the B subgenome exhibited fewer genes and lower diversity in the *TPS* and *NB-ARC* families. These findings indicate that BB may have originated from an unfound or extinct species within the *Perilla* spp. The BB donor might be less diversified than AA, possibly adapting to a narrow geographic and climatic range.

## 1. Introduction

*Perilla frutescens* is a highly diverse plant species, which is widely cultivated and utilized across East Asia, Southeast Asia, and other regions as an important industrial crop. Wild *Perilla* spp. has been discovered in East Asian countries such as China and Japan. It is generally believed that *P. frutescens* was first domesticated in China [1,2,3]. Nowadays, it has been introduced to Europe and North America, mainly as a spice plant. *P. frutescens* serves as both a vegetable for consumption and as a spice due to its aromatic properties. It also boasts a long history of medicinal use. Its unique therapeutic value is documented in the classical Chinese medical text, the Compendium of Materia Medica, and the leaves, stems, and seeds of *P. frutescens* are listed in the Chinese Pharmacopoeia. Behind its extensive applications and diversity lies a complex genomic makeup.

The identification of the karyotype of *P. frutescens* dates back to 1994, when it was recognized as an allotetraploid (2n = 4x = 40, AABB) [4]. Since then, several studies have explored the diploid progenitors of its two subgenomes. Ito et al. published the first report on its donor AA. By creating hybrid lines and combining karyotype identification methods, they found that the allotetraploid *P. frutescens* had ten common chromosomes with the diploid *Perilla citriodora* in the triploidy F1 generation, so they believed that *P. citriodora* was one donor of *P. frutescens* [4]. Subsequently, Ito et al. provided further evidence indicating *P. citriodora* as a donor of *P. frutescens* from the perspective of key terpene synthases (TPSs). They found that the sequences of geraniol synthases from *P. citriodora* and *P. frutescens* were mostly the same [5]. In 2021, a study of the first *P. frutescens* and *P. citriodora* genomes confirmed that *P. citriodora* is the AA donor of *P. frutescens* at the genomic level [6].

Although the origin of the A subgenome in *P. frutescens* has gradually become clear, the other donor, BB, remains enigmatic. To date, no germplasm resources have been confirmed as direct descendants of BB. This has severely hampered the deep understanding of the entire history of this widely used crop, including its origin, evolution, as well as the mechanisms of its genomic evolution [4,6,7,8,9,10].

As a species rich in chemical diversity, *P. frutescens* was found to be classifiable into distinct germplasm groups based on the major compounds of its volatile oils. This led to the introduction of the concept of chemotypes, establishing a framework for understanding its metabolic diversity [11]. Chemotypes of leaves with different monoterpene components exhibit distinct odors, endowing them with entirely different sensory characteristics and application values. However, the chemotypes of *P. frutescens* are different from the diploid *P. citriodora*. In numerous germplasm surveys, the perillaldehyde type and perilla ketone type have been found to be comparatively common [12,13,14]. In contrast, in *P. citriodora*, the citral type currently exhibits the highest prevalence, and no evidence of the perillaldehyde type has been found in this diploid species [4,13,15]. This difference in chemotype distribution suggests that the emergence of the perillaldehyde type may be closely associated with the contribution of another unknown donor, BB. Since the BB germplasm has not been discovered yet, the direct evidence supporting this hypothesis is still lacking, making it an unresolved issue in understanding the formation of chemotype differences and the metabolic evolution of *P. frutescens* [4,9,10,15]. Thus, revealing the characteristics of the BB genome will also be important for the exploration of this species’ metabolic evolution.

Allopolyploids are relatively common in plants, but it is not an easy task to identify the donor species of these subgenomes. With the development of sequencing technology, genomic analyses such as collinearity, phylogenetics, structural variation, and gene structures of homologous genes have become important methods for inferring the taxonomic status and potential characteristics of the donor species [16,17]. These methods are suitable choices for investigating *P. frutescens* to make inferences about its genomes. In addition, because perillaldehyde is a monoterpene, it is evident that the TPSs determining the biosynthesis of perillaldehyde hold significant importance for studying the evolution of *P. frutescens* and identifying clues for BB. Previous studies have also proved that the evolutionary history of *P. frutescens* can be inferred by the sequence similarity of terpenoid synthases [5,8]. Therefore, systematically analyzing the diversity of *TPS*s in *P. frutescens* and their subgenomic distributions could provide critical points for elucidating the evolutionary origins of BB and its related chemotypes. The published genomes of the *Perilla* spp. provide genomic insights for studying BB, enabling the inference of potential characteristics possessed by BB at the genomic level.

Against this background, we aimed to infer the characteristics of the unknown ancestor, BB, of *P. frutescens* through multi-dimensional analyses of its genomic information. We compared the protein sequence and gene structural similarity of the B subgenome of *P. frutescens* with the A subgenome and the genomes of two close relatives of the *Perilla* spp. Furthermore, a set of “BB progenitor-specific genes” was screened and used to predict potential BB traits. We also analyzed the distribution of the *TPS* gene family and nucleotide-binding adaptor shared by Apaf-1, R proteins, and the Ced-4 (*NB-ARC*) disease resistance gene family in each subgenome. The results of these multi-dimensional analyses were used to construct inferences about the unknown BB progenitor of *P. frutescens* at the genomic level and propose hypotheses regarding its potential geographical distribution.

## 2. Results

### 2.1. Analysis of Protein Sequence Similarity Between BB and Its Related Species

To explore whether BB might have originated from a species of the genus *Perilla* or some other closely related genus, we first dissected PF40, a genome of *P. frutescens* with clearly defined A and B subgenomic regions [6]. The genome analysis revealed that the A subgenome showed an advantage in terms of the number of genes, with a total of 20,936 genes, while the B subgenome had 17,676 genes [6]. We selected the A subgenome, which was obtained by splitting PF40, as well as two other species, *Lavandula angustifolia* and *Ocimum basilicum,* from the genera *Lavandula* and *Ocimum* of the Family Labiatae. These two species have high-quality reference genome data and are known to be the closest evolutionary relatives of *P. frutescens* [18,19,20,21].

**Figure 1:**
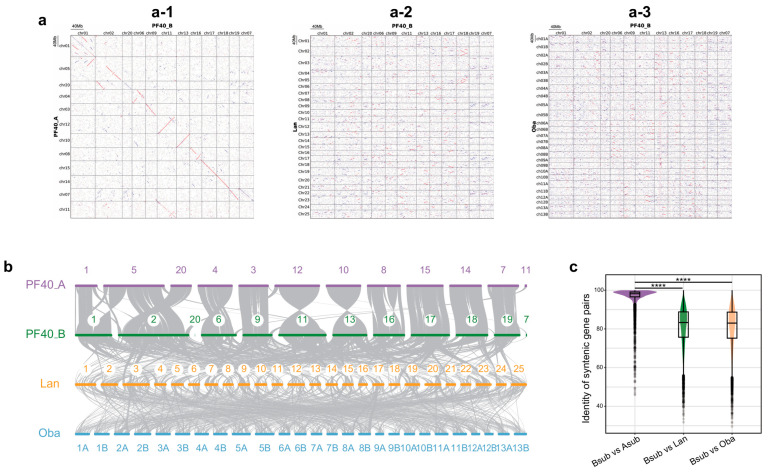
Collinearity among the B subgenome, the A subgenome, and the two closely related species. (**a**) Similarity between the B and A subgenomes of *P. frutescens*, *L. angustifolia,* and *O. basilicum* based on their protein sequences. Each red dot represents the high similarity of the proteins encoded by the corresponding syntenic genes from the two genomes. (**a-1**–**a-3**) show the genomic dot plots between the B subgenome and the A subgenome, the B subgenome and the *L. angustifolia* genome, and the B subgenome and the *O. basilicum* genome, respectively; (**b**) Synteny between the B subgenome and the A subgenome of *P. frutescens*, as well as the genomes of *L. angustifolia* and *O. basilicum*. The gray lines connect the genes that have the most similar sequences. PF40_A represents the A subgenome of *P. frutescens*, PF40_B represents the B subgenome of *P. frutescens*, Lan represents the genome of *L. angustifolia*, and Oba represents the genome of *O. basilicum*; (**c**) Distribution for the identities of the protein sequences of syntenic gene pairs (two-tailed Student’s *t*-test, **** *p* < 0.0001). These gene pairs were obtained through analysis of the synteny in (**b**).

Next, the protein sequences were aligned between the split B subgenome against each of the other three genomes. Synteny analysis based on sequence similarity indicated that the B subgenome and the A subgenome exhibited strong collinearity in all 10 chromosomes, suggesting a high degree of homology in the sequences of the coding genes (Figure 1a–c). However, significant reductions in collinearity were observed when the B subgenomes were analyzed in the two close relatives, *L. angustifolia* and *O. basilicum* (Figure 1a–c). A similar high collinearity between subgenomes and low collinearity between the subgenomes and the genomes of close relatives had also been observed in a previous study of *O. basilicum* [21]. Although the B subgenome shares many homologous sequence fragments with *L. angustifolia* and *O. basilicum*, these fragments were much shorter and more scattered (Figure 1a,b). The lack of genomic data for more species of the Lamiaceae family limits the systematic analysis of their genomic similarities. However, the highly conserved sequences of the A and B subgenomes suggest that BB may have originated from a species of the genus *Perilla* or even the same ancestor species as AA.

### 2.2. Analysis of Similarity of Gene Structures Between BB and Its Related Species

**Figure 2:**
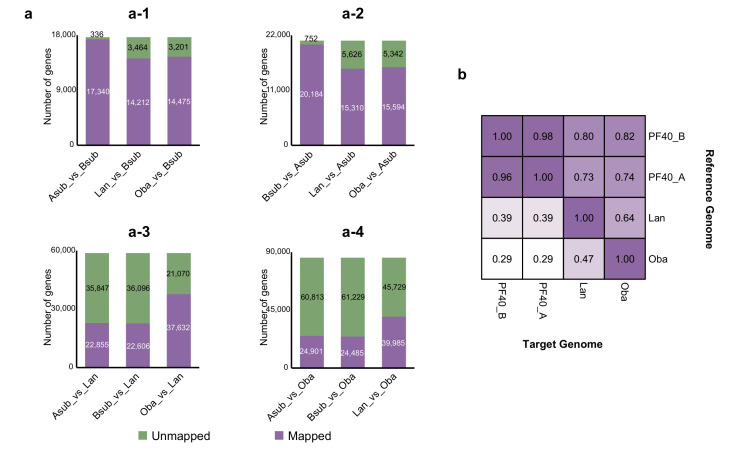
Pairwise comparison of the B subgenome, the A subgenome, and two closely related genomes based on the similarity of the gene structures of homologous genes. (**a**) Statistics of genes with similar structures in the B subgenome and other genomes. “Mapped” represents genes with similar structures and is represented by purple bars, while “Unmapped” represents genes with dissimilar structures and is represented by green bars. (**a-1**–**a-4**) show the number of genes that are structurally similar to other genomes in the B subgenome, A subgenome, and the genomes of *L. angustifolia* and *O. basilicum,* respectively. (**b**) Proportion of genes in the reference genome that are structurally similar to homologous genes in the target genomes. Asub represents the A subgenome of *P. frutescens*, Bsub represents the B subgenome of *P. frutescens*, Lan represents the genome of *L. angustifolia*, and Oba represents the genome of *O. basilicum*.

The protein sequence similarity can only partly reflect the relationship between homologous genes. The number of exons, the splicing sites, and other gene structures can also be used to evaluate the similarities between homologous genes [22] and should be considered as well. So we further compared the B subgenome, the A subgenome, *L. angustifolia,* and *O. basilicum* with each other based on the gene structures of homologous genes. The results showed that when the B subgenome was used as the reference genome, the A subgenome had the largest number of similar genes with it (Figure 2a). When the A subgenome was used as the reference genome, the B subgenome also had the largest number of similar genes with A, while the similarity of either A or B with *L. angustifolia* or *O. basilicum* was significantly lower (Figure 2a,b). Given the similarities in both the protein sequences and gene structures, it is highly likely that BB also originated from a *Perilla* species, but it still maintains a certain degree of differentiation from AA.

### 2.3. Screening and Functional Annotation of BB Progenitor-Specific Genes

**Figure 3:**
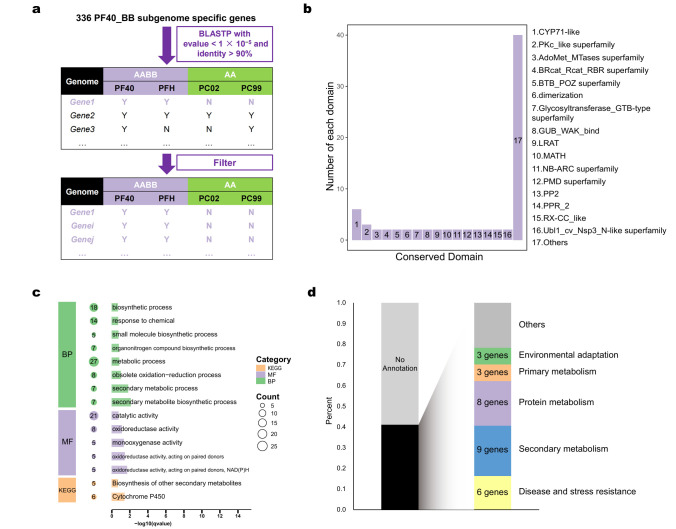
Screening and functional annotation of BB progenitor-specific genes. (**a**) Screening methods for “BB progenitor-specific genes” based on the published genomes of *Perilla* spp. (**b**) Conversed domain prediction of the “BB progenitor-specific genes”. “Conserved Domain” represents the names of conserved domains. “Number of each domain” indicates the number of each type of conserved domain included in “BB progenitor-specific genes”. CYP71-like: Cytochrome P450 family 71-like. PKc_like superfamily: Protein Kinases, catalytic domain. AdoMet_MTases superfamily: S-adenosylmethionine-dependent methyltransferases. BRcat_Rcat_RBR superfamily: Benign-catalytic and required-for-catalysis domains, part of the RBR (RING1-BRcat-Rcat) domain. BTB_POZ superfamily: Broad-Complex, Tramtrack and Bric a brac/poxvirus and zinc finger. GUB_WAK_bind: Wall-associated receptor kinase galacturonan-binding. LRAT: Lecithin retinol acyltransferase. MATH: Meprin and TRAF-C homology. NB-ARC superfamily: Nucleotide-Binding adaptor shared by APAF1, certain R genes and CED4. PMD superfamily: Plant mobile domain. PP2: Phloem protein 2. PPR_2: Pentatricopeptide repeat family. RX-CC_like: Coiled-coil domain of the potato virux X resistance protein and similar proteins. Ubl1_cv_Nsp3_N-like superfamily: First ubiquitin-like domain located at the N-terminus of coronavirus SARS-CoV non-structural protein 3 and related proteins; (**c**) GO and KEGG enrichment analysis of the “BB progenitor-specific genes”. BP represents “Biological process”, MF represents “Molecular function”; (**d**) Classification of functional annotation for “BB progenitor-specific genes” based on homogenes.

In order to speculate on the characteristics of the BB progenitor, we further focused on the BB progenitor-specific genes. We systematically compared the protein sequences and gene structures of homologous genes between the B and A subgenomes. This approach identified 336 genes that are specific to the B subgenome (Figure 2a, Supplementary Table S1). To obtain more convincing BB progenitor-specific genes, our further analysis included the two published allotetraploid *P. frutescens* genomes, PF40 and Hoko-3, as well as the two diploid *P. citriodora* genomes, PC02 and PC99, which were both considered to be the AA diploids. We retained genes that were present in both PF40 and Hoko-3 but were absent in both PC02 and PC99. Eventually, 90 genes were obtained, which we defined as “BB progenitor-specific genes” (Figure 3a). Due to the extremely similar genomes, there may exist genes that are either present or absent in both subgenomes, which would cause a deviation in the screening of “BB progenitor-specific genes”. There are significant differences in geographical distribution, as well as morphological and genomic differences, between PF40 and Hoko-3 [6,23], and the genome size of PC99 is 10% smaller than that of PC02 [6]. This diversity of genomes can, to some extent, enhance the reliability of the screening for “BB progenitor-specific genes”.

The annotation of the BB progenitor-specific genes was then performed by three different methods. As shown in Figure 3b, the proteins encoded by the 90 genes contain a total of 77 complete domains. Among the 90 genes, the cytochrome P450 family 71 (CYP71-like) domain is the most numerous. The CYP71 family genes are involved in secondary metabolic processes in many species of Lamiaceae, and they have been found to modify monoterpenoids in plants such as mint and perilla [24,25,26]. Other domains have not been clearly reported to be related to the secondary metabolisms of perilla and its related species. Therefore, we speculate that BB may have different monoterpenes from AA.

GO and KEGG enrichment showed that 90 “BB progenitor-specific genes” were mainly enriched in secondary metabolic processes and chemical responses. This suggests that these genes may be related to specific environmental adaptations (Figure 3c).

Then, we annotated each of the 90 genes based on the results of blasting in NCBI and searching for the functions of the identified homologous proteins in the Uniprot database. The results showed that 32.2% of the genes were related to secondary metabolism, disease and stress resistance, and protein and fatty acid metabolism functions (Figure 3d, Supplementary Table S2). Among them, TPS KAH6789932.1 caught our attention. BLAST v2.10.1 analysis revealed that this protein shares 52.76% sequence identity with the SabS1 enzyme from *Salvia pomifera*, which has been reported to synthesize sabinene (Supplementary Table S2) [27]. In addition, we identified KAH6788468.1 as a homolog of the late blight resistance protein R1A-10 in *Solanum demissum* through a BLAST alignment, which showed 32.62% sequence identity (Supplementary Table S2). Since the sequence similarity between KAH6788468.1 and R1A-10 is relatively low, it is likely to have a completely different function from R1A-10. The domain prediction indicates that KAH6788468.1 contains a complete NB-ARC domain, so it may be related to disease resistance [28] (Figure 3b, Supplementary Table S3). So, we speculated that BB may have originated in an area with a high incidence of late blight disease.

### 2.4. Asymmetric Distribution of TPS Families Between Subgenomes

**Figure 4:**
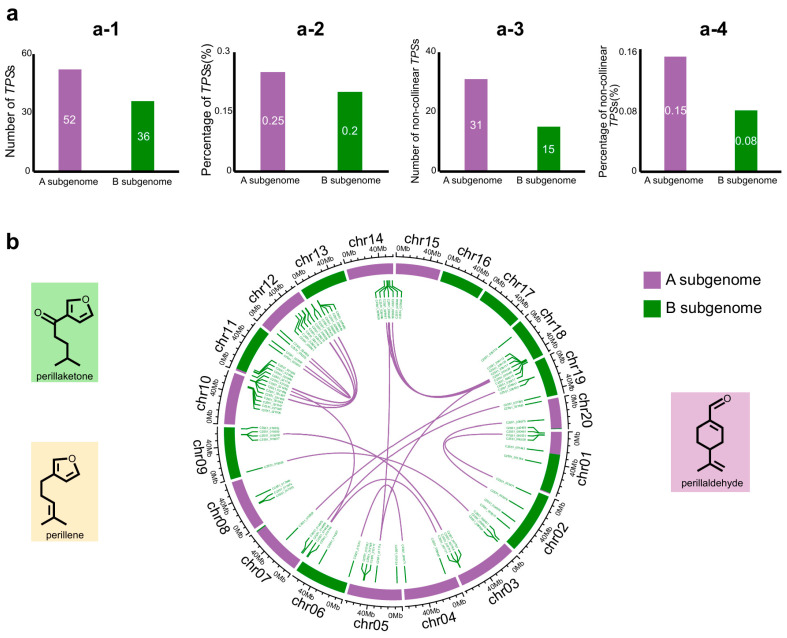
Asymmetric distribution showed that the *TPS* family in the B subgenome has fewer genes and non-collinear genes. (**a**) Comparison of *TPS*s located in the A subgenome and the B subgenome. (**a-1**) shows the number of *TPS*s in the B subgenome and A subgenome. (**a-2**) shows the percentage of *TPS*s in total genes of the B subgenome or A subgenome. (**a-3**) shows the number of non-collinear *TPS*s in the B subgenome or A subgenome. (**a-4**) shows the percentage of non-collinear *TPS*s in the B subgenome or A subgenome. (**b**) Distribution and collinearity of *TPS*s in the B and A subgenomes. Purple lines connect genes with highly similar protein sequences.

Given that a TPS is included in the “BB progenitor-specific genes” and the number of CYP71-like domains in the “BB progenitor-specific genes” is the highest, we speculate that BB may differ from AA in terms of monoterpene diversity. Considering that TPSs determine the formation of the terpene skeleton and affect the diversity of the terpene skeleton, and as cytochrome P450 modifies terpenes [25], the diversity of monoterpene chemotypes in *P. frutescens* might be the result of both terpene synthases (TPSs) and cytochrome P450 (CYP450). The TPS family is closely related to the chemical diversity of *P. frutescens*, and its distribution and functional differentiation can reflect the differences in origin among subgenomes. Firstly, we identified 99 candidate *TPS*s from the PF40 genome by HMMER [29]. Using CD-Search, we further screened the corresponding proteins with complete Terpene_cyclase_plant_C1, the PLN02592 superfamily, the PLN02279 superfamily, or the Isoprenoid_Biosyn_C1 superfamily domain. Finally, 88 *TPS*s were obtained for subsequent analysis. The gene distribution showed that 52 genes were located in the A subgenome and 36 genes were located in the B subgenome (Figure 4a).

In terms of the proportion of all genes in the subgenome, the proportion of *TPS*s in the A subgenome is 0.25%, and in the B subgenome, it is 0.20%. The *TPS*s in the A subgenome have a greater quantitative advantage (Figure 4a). Analysis based on protein sequence similarity revealed that there were 21 pairs of *TPS* genes that were colinear between the A subgenome and the B subgenome, accounting for 40.4% and 58.3% of the total *TPS*s in the A subgenome and the B subgenome, respectively (Figure 4a,b). All TPS genes except the 21 pairs of collinear *TPS*s were defined as non-collinear *TPS*s (Figure 4a). This indicates that the A subgenome has more specific *TPS*s. The quantitative and differentiative advantages of the *TPSs* in the A subgenome also indicate that the A subgenome may make a greater contribution to the terpene diversity of *P. frutescens*. It also suggests that although the A and B subgenomes are highly similar, there is still differentiation between the BB-donor species and *P. citriodora* that may have led to their chemotypic differences. The species contributing to the B subgenome might have had different traits from *P. citriodora* in the past.

### 2.5. Asymmetric Distribution of NB-ARC Families Between Subgenomes

*P. frutescens* is an important economic crop, so studying its disease resistance and control has significance for its production. Its resistance to different diseases can also reflect the *P.* *frutescens*’ adaptability to its environment. It is widely believed that the proteins involved in pathogen recognition in plants are resistance (R) proteins [30]. Most of these R proteins contain a central nucleotide-binding domain (NB-ARC), and this domain is believed to regulate the activity of R proteins [28]. Therefore, studying the differences in the *NB-AR*C genes in these subgenomes is beneficial for inferring the resistance characteristics and geographical distributions of the donors of *P. frutescens*.

**Figure 5:**
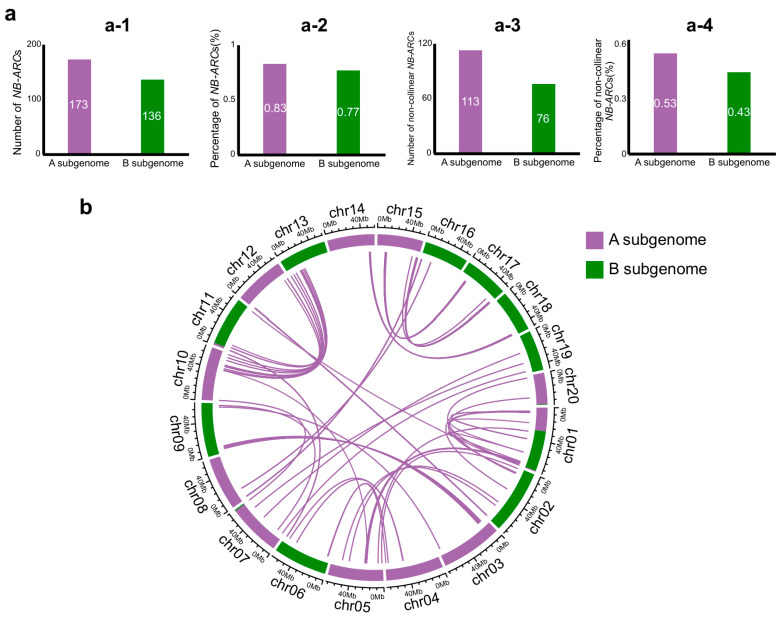
Asymmetric distribution showed the *NB-ARC* family in the B subgenome has fewer genes and more non-collinear genes. (**a**) Comparison of *NB-ARC*s located in the A subgenome and the B subgenome. (**a-1**) shows the number of *NB-ARC*s in the B subgenome or A subgenome. (**a-2**) shows the percentage of *NB-ARC*s in the total genes of the B subgenome or A subgenome. (**a-3**) shows the number of non-collinear *NB-ARC*s in the B subgenome or A subgenome. (**a-4**) shows the percentage of non-collinear *NB-ARC*s in the B subgenome or A subgenome. (**b**) Collinearity of *NB-ARC*s in the B and A subgenomes. Purple lines connect genes with highly similar protein sequences.

A gene containing the NB-ARC domain and sharing 32.62% protein sequence identity with R1A-10 was identified among the “BB progenitor-specific genes” (Supplementary Tables S2 and S3). This finding suggests that the disease-resistant characteristics of BB warrant further attention. These characteristics might reflect the differences in evolution between BB and AA. Therefore, we identified and analyzed the *NB-ARC*s in the PF40 genome. Through a search of HMMER, a total of 355 candidate *NB-ARC*s were identified in PF40. Using CD-Search, we further screened the corresponding proteins for the complete NB-ARC domain. Eventually, 309 *NB-ARC*s were obtained for subsequent analysis. Among them, 136 *NB-ARC*s were distributed in the B-subgenomic region, accounting for 0.77% of the total genes in the B subgenome, and 173 *NB-ARC*s were distributed in the A subgenome, accounting for 0.83% of the total genes in the A subgenome (Figure 5a). There are a total of 60 pairs of co-linear *NB-ARC*s in the A and B subgenomes, accounting for 34.7% and 44.1% of the total *NB-ARC*s in the A and B subgenomes, respectively (Figure 5a,b). All *NB-ARC* genes except the 60 pairs of collinear *NB-ARC*s were defined as non-collinear *NB-ARC*s (Figure 5a).

Thus, we found that the A subgenome has more *NB-ARC*s compared to the B subgenome, and its gene family diversity is higher. This genomic feature suggests that the AA donor may have evolved with more complex and diverse disease resistance mechanisms, and this difference may reflect that the AA and BB donors experienced different pathogenic selection pressures during their evolution: the AA donor had a broader adaptability, while the BB donor may have adapted to specific habitats with relatively lower pathogenic pressure.

## 3. Discussion

It is generally believed that *P. frutescens* is an allotetraploid (2n = 4x = 40, AABB). The diploid progenitor of the A subgenome has been identified. However, the origin of the B subgenome remains unclear. Since the A and B have fused in several chromosomes [6], it is hard to determine the exact chromosome number of BB. Might it originate from another species in the same genus? Or is there a possibility that the BB subgenome comes from a species of a closely related genus? In this study, based on the published genomes of *Perilla* spp., *L. angustifolia,* and *O. basilicum*, we discovered that, compared with the two species from other genera, the A subgenome was highly similar to the B subgenome in their protein sequences and conserved gene structures of homologous genes. Furthermore, we analyzed the “BB progenitor-specific genes” and found an asymmetric distribution of genes from both the *TPS* and *NB-ARC* families in the A and B subgenomes. These genes show a higher number and diversity in the A subgenome.

Previous speculations about the progenitor of the B subgenome have suggested that it existed in China, but they have failed to identify the species from which this subgenome originated [9]. Genomic analysis indicates that the B subgenome is older than the A subgenome and its donor, *P. citriodora*, and that the B subgenome shows great concordance with the genome of *P. citriodora* [6]. However, no in-depth analysis or feature inference has been reported in the B subgenome. This study was the first to focus on the taxonomic status and key genetic characteristics of the B subgenome. Based on the above analysis, we infer that the unfound donor of the B subgenome of *P. frutescens* might have been a species within the genus *Perilla* that adapted to a specific habitat. This study has reduced the possibility of the BB’s origin from other genera and so has narrowed down the search range to the genus *Perilla*.

Genomic analysis holds significant application value in exploring the origin and history of polyploids. This study evaluated the similarity between the B subgenome and its relatives by comparing the collinearity of their protein sequences and the structural similarity of their homologous genes. Through the screening of “BB progenitor-specific genes” and the analysis of gene families, the possible characteristics of BB were speculated upon. Similarly, genomic methods have also been applied to other polyploids. For example, for *Avena barbata* (AABB), researchers selected 12 closely related species of the same genus to compare and inferred the unknown donors of the subgenomes through phylogenetic and sequence similarity analyses. They also compared subgenome-specific “PAV genes” and further analyzed the functions of these genes [16]. In *Elymus nutans* (StStYYHH), researchers compared the degree of similarity and the evolutionary relationship between the subgenome and 28 related species through phylogenetic analysis and sequence similarity studies, and inferred the taxonomic status of the unknown Y subgenome [17]. There were also some different approaches to discriminate between subgenomes. For example, in *Trapa*, by collecting population samples of 26 species or varieties within the genus, researchers clarified the evolution of subgenomes among different species and varieties through phenotypic measurement, population structure analysis, and population differentiation analysis, and discovered that one species within the genus might contain a new subgenome [31]. In conclusion, our future work could focus on collecting as many *Perilla* species as possible for phylogenetic and comparative genomic analysis, and the analyses of subgenomic characteristics at the expression level could also be included.

We have discovered that the protein sequences and gene structures of the B subgenome are highly conserved with the A subgenome. However, the most closely related species, *Keiskea szechuanensis* [19], cannot be included in the comparison due to a lack of genomic data. Furthermore, the genomes of only four species of the genus *Perilla* have been sequenced. We defined the “BB progenitor-specific genes” based on their presence/absence patterns in *P. frutescens* and *P. citriodora*, namely, that they exist in the two genomes of *P. frutescens* (PF40 and Hoko-3) but are absent in the two genomes of *P. citriodora* (PC02 and PC99). Thus, the structural variations that are common to both of the two *P. frutescens* genomes or the two *P. citriodora* genomes may affect the determination of the “BB progenitor-specific genes”. The annotation bias of any one of the genomes may also have led to false positives and false negatives among the “BB progenitor-specific genes”. The newly generated genes in the PF40 and Hoko-3 genomes, which might be produced by tandem repeats, fragment repeats, or other mutations, may have resulted in the new genes generated through replication after polyploidization being wrongly identified as “BB progenitor-specific genes”. If more available genomes of related species of *P. frutescens,* in or out of the genus *Perilla*, can be obtained, the screening for PAV as “BB progenitor-specific genes” will be able to be analyzed from the perspective of the pan-genome, and the prediction results will be more accurate. In addition, due to the lack of possible BB progenitor samples, reliable experiments cannot be performed to verify our hypotheses. It is also difficult to confirm whether the donor species of BB has become extinct. This makes it impossible to obtain more information about BB at present. Future work can further explore possible samples from large-scale wild investigations in the targeted area. Then, the genomic and population genomics can be combined to deepen our understanding of BB and the evolution and domestication history of *P. frutescens.*

In conclusion, this study provides evidence from a comparative genomic perspective to infer the taxonomic status and potential distribution of BB, which can help search for and conduct an in-depth exploration of the undiscovered BB ancestor.

## 4. Materials and Methods

### 4.1. Preparation of Genomic Data

The PF40 genome data of *Perilla frutescens* were downloaded from the Genomes database of NCBI (https://ftp.ncbi.nlm.nih.gov/genomes/all/GCA/019/511/825/GCA_019511825.2_ICMM_Pfru_2.0/ (accessed on 18 July 2025)). The *Lavandula angustifolia* genome data were from NCBI (https://ftp.ncbi.nlm.nih.gov/genomes/all/GCA/028/984/105/GCA_028984105.1_UGA_lav_v1/ (accessed on 20 July 2025)). The *Ocimum basilicum* genome data were obtained from the BasilBase website (https://basil.breedbase.org/easy_gdb/about.php (accessed on 25 August 2025)).

### 4.2. Collinearity Analysis

Before collinear analysis, the *Perilla frutescens* PF40 genome was split using SAMtools (v1.10) [32] based on subgenomic region information to obtain the AA and BB subgenomic sequences. The collinearity dot map of the genome was plotted using WGDI (v0.6.5) [33]. The parameters of the WGDI dot plot configuration file were set as multiple = 1, score = 100, evalue = 1 × 10^−5^, repeat_number = 10, position = order, blast_reverse = false. Chromosome synteny plots were drawn using the JCVI (v1.1.11) [34]. The JCVI parameters were set as the default parameters. The syntenic gene pairs generated by JCVI were further processed by BLASTP to calculate sequence identity.

### 4.3. Analysis of Gene Structural Similarity

The intersect tool of BEDtools (v2.30.0) [35] was used to split the PF40 genome annotation gff file into gff files for the A and B subgenomes. Liftoff (v1.6.3) [22] was used to analyze the gene structure similarity between genomes based on the gff annotation files of genomes, with parameters of Liftoff set to default.

### 4.4. Enrichment Analysis and Annotation of Genes

GO and KEGG enrichment analysis of the gene set used EggNOG-mapper (v6.0.5) [36] and TBtools (v2.376) [37]. For each gene in the gene set, we searched for the first homologous gene in the uniprot_sprot database that met the standard of evalue > 1 × 10^−5^ through BLASTP [38], and took it as the preliminary annotation of each gene in the gene set. Then we extracted the detailed functional descriptions of these homologous genes in the Uniprot [39] database as further functional annotations.

Batch CD-Search [40] (https://www.ncbi.nlm.nih.gov/Structure/bwrpsb/bwrpsb.cgi (accessed on 12 September 2025)) was used for the classification of gene families. The protein sequences corresponding to the gene set were used as input. The domains in the Batch CD-Search results with incomplete N or C termini or evalue > 1 × 10^−5^ were removed. The information of the filtered domains was statistically analyzed using a custom script and visualized using the R package “ggplot2” (v3.5.0) (https://ggplot2.tidyverse.org (accessed on 25 September 2025)).

### 4.5. Identification and Collinearity Analysis of the TPS Family

To identify candidate TPS genes in *P*. *frutescens*, the Hidden Markov Model (HMM) profile corresponding to the Terpene_synth domain (PF01397) and Terpene_synth_C domain (PF03936) was retrieved from Pfam (http://pfam.xfam.org/ (accessed on 9 July 2025)). Using “hmmsearch --cut_tc” of HMMER (v3.3.2) (http://hmmer.org/ (accessed on 10 July 2025)), we searched for sequences with an evalue < 1 × 10^−5^ in the protein sequences of the PF40 genome based on the two HMM files, then merged and removed duplicates. The collinearity analysis of the *TPS*s in PF40 was conducted using diamond (v2.0.11.149) [41] and TBtools. Distribution and collinearity of genes were visualized using the R package “circlize” [42].

### 4.6. Identification and Collinearity Analysis of the NB-ARC Family

To identify candidate *NB-ARC*s in *P*. *frutescens*, the Hidden Markov Model (HMM) profile corresponding to the NB-ARC domain (PF00931) was retrieved from Pfam (http://pfam.xfam.org/ (accessed on 5 September 2025)). The following analyses were conducted with the same method as for the TPS family.

## Data Availability

Data are contained within the article or Supplementary Materials.

## Supplementary Materials

The following supporting information can be downloaded at: https://www.mdpi.com/article/10.3390/plants14233698/s1, Table S1: 336 genes that are specific to the B subgenome based on the similarity in protein sequences and gene structures; Table S2: Annotation of “BB progenitor-specific genes” through the Uniprot database based on homologous genes; Table S3: Conserved domains of the BB progenitor-specific genes that were predicted through CD-search.
